# Deciphering tuberculosis: lysosome-centric insights into pathogenesis and therapies

**DOI:** 10.3389/fcimb.2025.1582037

**Published:** 2025-05-14

**Authors:** Cui Bao, Yuanyuan Zhang, Jiao Feng, Xiuwen Hong, Nan Gao, Ganzhu Feng

**Affiliations:** ^1^ Department of Respiratory and Critical Care Medicine, The Second Affiliated Hospital of Nanjing Medical University, Nanjing, Jiangsu, China; ^2^ Department of Respiratory and Critical Care Medicine, The Second Clinical Medical School of Nanjing Medical University, Nanjing, Jiangsu, China

**Keywords:** *Mycobacterium tuberculosis* (Mtb), lysosomes, interaction, mechanism, treatment

## Abstract

Tuberculosis is a widely spread disease caused by *Mycobacterium tuberculosis* (Mtb). The pathogenicity of the pathogen is closely associated with the immune defense mechanisms of the host cells. As key cellular degradation and metabolic centers, lysosomes critically regulate tuberculosis infection. When Mtb invades the host, it is taken up by macrophages and enters phagosomes. Subsequently, the phagosomes fuse with lysosomes and form phagolysosomes, which eliminate the pathogenic bacteria through the acidic environment and hydrolytic enzymes within lysosomes. However, Mtb can interfere with the normal functions of lysosomes through various strategies. It can secrete specific factors (such as ESAT-6, ppk-1, and AcpM) to inhibit the acidification of lysosomes, enzyme activity, and the fusion of phagosomes and lysosomes, thereby enabling Mtb proliferation within host cells. An in-depth exploration of the mechanism of the interaction between Mtb and lysosomes will both uncover bacterial immune evasion strategies and identify novel anti-tuberculosis therapeutic targets.

## 1 Introduction

Tuberculosis is mainly caused by the intracellular pathogen *Mycobacterium tuberculosis* (Mtb). It most commonly infects the lungs but can also affect other organs, including the intestines and bones. It seriously threatens human health and poses a heavy burden on global health services. According to the “Global Tuberculosis Report 2024” released by the World Health Organization (WHO), it was estimated that 10.8 million people worldwide suffered from TB and approximately 400,000 had drug-resistant tuberculosis (DR-TB); additionally, the disease caused 1.25 million deaths globally (Organization, W.H, 2023). Factors such as poverty, malnutrition, HIV infection, smoking, diabetes, chemotherapy, and weakened immunity all significantly increase individuals’ susceptibility to infections (Chai et al., 2018; Luies and du Preez, 2020). Fortunately, TB is a preventable and generally curable disease, and currently, the first-line drugs for TB treatment include isoniazid, rifampicin, pyrazinamide, ethambutol, and streptomycin (Zumla et al., 2013). However, the emergence of multidrug-resistant tuberculosis (MDR-TB) has presented new challenges to the treatment of TB. Thus, in-depth study of the interaction between Mtb infection and the host is conducive to our exploration of new treatment approaches.

Lysosomes are organelles discovered by de Duve in 1955 through specific enzyme staining with the aid of light microscopes and electron microscopes (de Duve, 2005). Lysosomes are acidic organelles encapsulated by a single membrane with a pH value of approximately 4.6. The endolysosomal system in cells consists of early endosomes (EE), recycling endosomes (RE), late endosomes (LE), and lysosomes (LY), and it is crucial for a variety of cellular functions, including membrane trafficking, protein transport, autophagy, and signal transduction (Luzio et al., 2007). The lysosomes contain a variety of hydrolases, including acid phosphatase, protease, lipase, and glycosidase, and the acidic interior optimally activates these hydrolases and facilitates the breakdown of intracellular waste and exogenous pathogens (Appelqvist et al., 2013). In addition, lysosomes can also eliminate pathogens through various pathways such as forming phagolysosomes by fusing with phagosomes, autophagy, and so on (Levine and Kroemer, 2008). As an organelle widely existing in eukaryotic cells, lysosomes not only degrade and recycle intracellular waste, organelles, and exogenous substances, but also regulate cell metabolism, mediate signal transduction, and participate in immune responses (Saftig and Klumperman, 2009).

**Figure 1:**
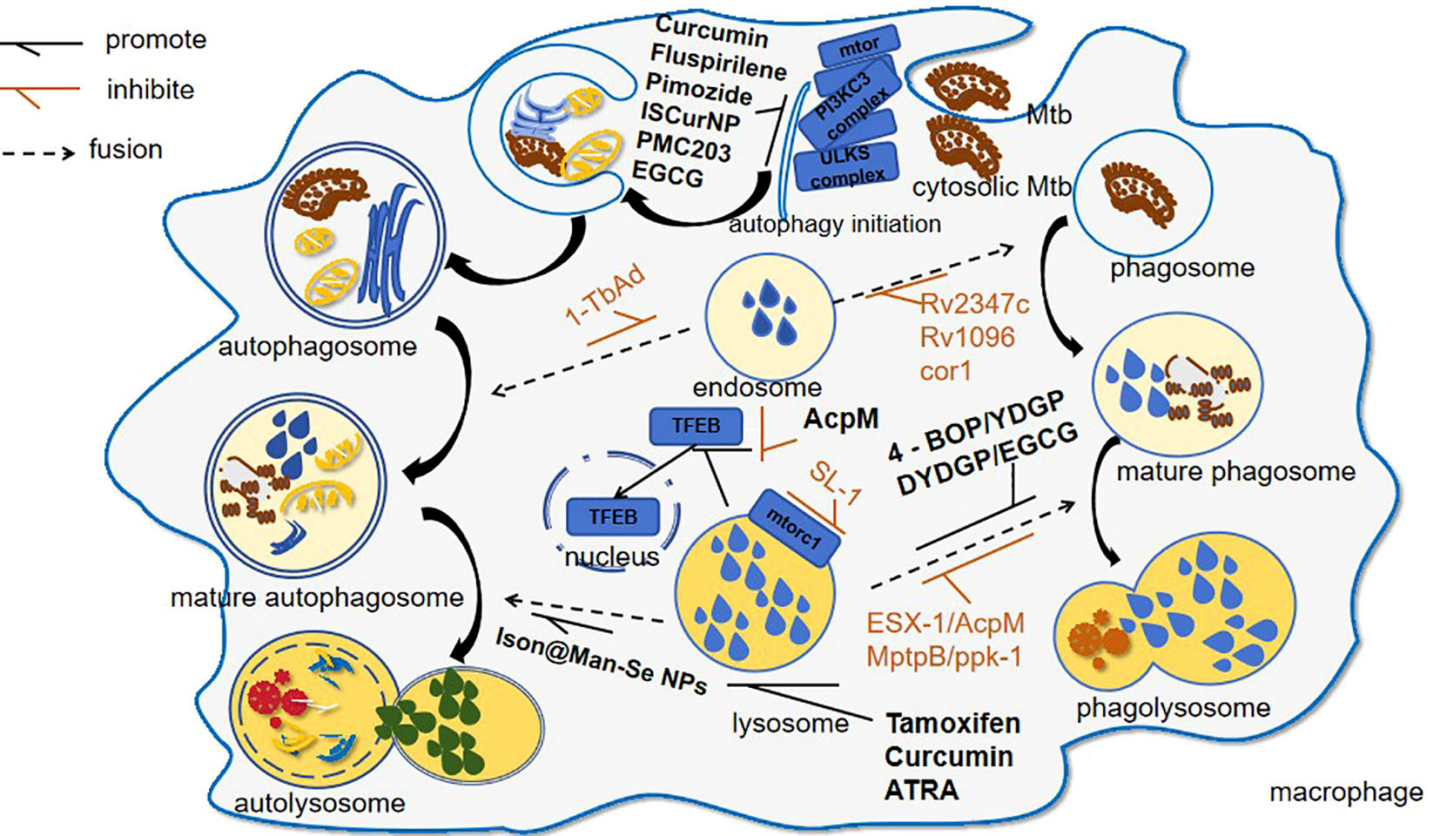
Mtb-induced lysosomal modulation and drug interventions following host invasion. This schematic delineates the autophagic and phagocytic pathways activated during Mtb infection: 1. Pathogen-Driven immune evasion: Mtb virulence factors, such as 1-TbAb, inhibit autophagosome maturation; 2. Therapeutic interventions: ① lysosomal potentiators like curcumin through upregulating lysosomal biogenesis; ② autophagy enhancers like Ison@Man-Se NPs through enhancing autophagosome–lysosome fusion.

When Mtb invades the body, macrophages serve as the first line of defense against its infection, swiftly recognizing and phagocytosing this pathogen (Russell, 2011). Meanwhile, lysosomes, as extremely crucial degradative organelles within cells, undertake the vital task of degrading pathogens within macrophages, playing a pivotal role in defending against Mtb infections (Armstrong and Hart, 1971). This review aims to delve deeply into the interaction between lysosome function and Mtb (show in **Figure 1**). Through in-depth exploration on the key role of lysosomes in Mtb, we deeply expect to provide a meaningful scientific theory for guiding anti-TB research, and thereby providing corresponding strategies for the treatment of TB.

## 2 The effect of Mtb infection on lysosomes

The Esx-1 secretion system (ESX-1), a specialized type VII secretion system (T7SS) unique to pathogenic mycobacteria including Mtb, is encoded by the genomic region of difference 1 (RD1) locus (Cole et al., 1998; Behr et al., 1999). On the one hand, ESX-1 secretes effector proteins, such as ESAT-6 and CFP-10. Both are immunodominant antigens and major virulence factors of Mtb, which disrupt the host cell membrane’s integrity to assist Mtb to more easily invade cells (Simeone et al., 2009). On the other hand, it can assist Mtb in surviving within cells by damaging phagosome membranes, preventing the fusion of autophagosomes with lysosomes and so on (Simeone et al., 2012) (Lienard et al., 2023). Transcriptomic and functional studies revealed that CD11c low monocyte-derived cells (MNC1) display compromised lysosomal activity (reduced TFEB expression, acidification, and proteolysis) relative to alveolar macrophages (AMs) and CD11c high counterparts (MNC2). Paradoxically, MNC1 exhibit superior capacity to harbor viable Mtb. This paradox is partly explained by Mtb’s ESX-1, which actively recruits lysosome-impaired MNC1 to lung tissue, creating protective niches for bacterial persistence. Targeting this host vulnerability, c-Abl inhibitors (e.g., nilotinib) restore lysosomal function via TFEB activation, proposing a novel host-directed therapy (HDT) to eliminate Mtb reservoirs (Zheng et al., 2024). Moreover, the Mtb Type VII secretion system effector Rv2347c employs a dual mechanism to subvert host defenses: it employs a dual mechanism to subvert host defenses, and it not only suppresses phagosome maturation by downregulating early markers (RAB5/EEA1), but also compromises lysosomal membrane integrity, ultimately facilitating bacterial escape from the degradative compartment (Jiang et al., 2024). MptpB, encoded by Rv0153c, as an effector molecule of the type VII secretion system of Mtb, is a key TB virulence factor with phosphoinositide phosphatase activity (Singh et al., 2005; Beresford et al., 2007). By dephosphorylating PI3P, it impedes phagosome maturation and lysosome–phagosome fusion, protecting Mtb from destruction (Beresford et al., 2007). On the basis of this, the MptpB inhibitor C13 enhances mycobacterial clearance through dual mechanisms: restructuring PI3P–phagosome interactions to promote phagolysosomal fusion, evidenced by increased LAMP-1 colocalization, and exhibiting synergistic bactericidal activity with frontline antibiotics (rifampin, bedaquiline, and pretomanid) through accelerated lysosomal trafficking and intracellular burden reduction (Rodríguez-Fernández et al., 2024). Similarly, AcpM, encoded by Rv2244, plays a crucial role in the biosynthetic pathway of the cell wall and closely related to the pathogenicity and drug resistance of Mtb (Miotto et al., 2022; Ling et al., 2024). It orchestrates immune evasion through dual posttranscriptional sabotage: miR-155-5p-mediated Akt-mTOR activation suppresses TFEB-driven lysosomal biogenesis, while coordinated repression of TFEB-regulated vesicular trafficking (e.g., LAMP1/RAB7) blocks phagolysosomal maturation, creating protected niches for Mtb persistence (Paik et al., 2022). Instead, the sulfolipid SL-1 of Mtb is encoded by Rv3821 and Rv3822 (Seeliger et al., 2012). It suppresses mTORC1 activity to promote TFEB nuclear translocation, thereby activating lysosomal biogenesis and potentiating lysosomal function. Intriguingly, SL-1 demonstrates complementary phagosomal acidification capacity, whereas SL-1-deficient mutants show impaired lysosomal trafficking concomitant with elevated intracellular bacterial survival rates (Sachdeva et al., 2020).

Apart from these, research has identified other influencers. For example, 1-TbAd, an abundant lipid in Mtb that is biosynthesized involving Rv3377c and Rv3378c, has been proven to be a naturally evolved phagolysosome disruptor (Mayfield et al., 2024) (Buter et al., 2019). It penetrates lysosomes, protonates, and concentrates within the acidic lysosomal environment, causing both lysosomal swelling and pH elevation. This cascade inactivates hydrolytic enzymes while hindering autophagosome maturation, thereby facilitating Mtb infection (Bedard et al., 2023). Beyond that, Rv1096 can inhibit the maturation of phagosomes, allowing Mtb to escape into the host cell cytoplasm (Deng et al., 2020). Additionally, PPK, which stands for “Polyphosphate kinase,” primarily includes ppk-1 (encoded by Rv2984) and ppk-2 (encoded by Rv3232c) (Singh et al., 2016). They critically regulate Mtb stress adaptation, enhancing resistance to thermal, acidic, oxidative, and hypoxic challenges while potentiating intramacrophage replication through polyP-mediated homeostasis (Cole et al., 1998; Sureka et al., 2007, Sureka et al., 2009; Singh et al., 2013). Chugh et al. (2024 PNAS) revealed polyphosphate kinase-1 (PPK-1) as a central metabolic regulator in Mtb pathogenesis through two complementary mechanisms: PPK-1 deficiency disrupts phthiocerol dimycocerosate (PDIM) biosynthesis to enhance phagolysosomal fusion-mediated bacterial clearance, while pharmacological inhibition of PPK-1 with raloxifene synergizes with frontline anti-TB drugs (isoniazid/bedaquiline) by subverting bacterial metabolic networks, collectively demonstrating the potential to shorten chemotherapy duration and combat MDR-TB through this dual host–pathogen targeting strategy (Chugh et al., 2024).

In addition to the factors aforementioned in TB, it is noteworthy that Coronin 1 (Cor1), a 57-kDa trimeric actin-binding protein essential for leukocyte cytoskeletal dynamics, emerges as a critical mediator of macrophage–Mtb interactions (Stocker et al., 2018). Its structural capacity to orchestrate actin remodeling becomes indispensable for pathogen containment during Mtb phagocytosis (Jayachandran et al., 2007; Bravo-Cordero et al., 2013). Mtb strategically hijacks Cor1 to orchestrate phagosomal arrest through a cAMP–cofilin1 signaling axis. Upon macrophage infection, Cor1 recruitment to phagosomal membranes coincides with cAMP elevation, which activates Slingshot phosphatase to dephosphorylate cofilin1. This cascade induces F-actin depolymerization, disrupting actin remodeling essential for phagosomal trafficking. Crucially, Cor1-mediated dual blockade of both actin cytoskeletal dynamics and vesicular fusion machinery creates protected compartments that evade lysosomal destruction (Saha et al., 2021). Mtb PknG is encoded by Rv0410c (Wang et al., 2021). It subverts host autophagy–lysosome fusion, impairs lysosomal acidification via V-ATPase inhibition, and destabilizes lysosomal membrane integrity, thereby hijacking autophagic flux to establish intracellular survival niches while evading lysosomal degradation, which underscores the therapeutic promise of dual-targeting PknG kinase activity and lysosomal functional restoration in TB treatment (Ge et al., 2022). The molecular mechanisms through which Mtb subverts host lysosomal function via key virulence determinants, along with corresponding therapeutic interventions, are systematically shown in (**Table 1**).

**Table 1:** The impact of Mtb pathogenic factors on lysosomes.

Virulence Factor	Encoding Genes	Mechanism of Action	Potential Therapeutic Targets
ESX-1 Secretion System	RD1 (Behr et al., 1999)	Secretes ESAT-6/CFP-10 to disrupt host membrane integrity (Simeone et al., 2009); damaging phagosome membranes, preventing the fusion of autophagosomes with lysosomes and so on (Simeone et al., 2012) (Lienard et al., 2023); ESX-1 recruits lysosome-impaired MNC1 to form protective niches; TFEB suppression reduces lysosomal biogenesis (Zheng et al., 2024).	Inhibitors of ESX-1 secretion; c-Abl inhibitors (e.g., nilotinib); lysosome-targeted nanoparticles.

Rv2347c (T7SS effector)	Rv2347c	Downregulates RAB5/EEA1 to block phagosome maturation; disrupts lysosomal membrane integrity (Jiang et al., 2024).	Small-molecule inhibitors of Rv2347c.
MptpB	Rv0153c (Singh et al., 2005)	Dephosphorylates PI3P to impair phagosome–lysosome fusion (Beresford et al., 2007).	MptpB inhibitor (e.g., C13) (Rodríguez-Fernández et al., 2024).
AcpM	Rv2244	Suppresses TFEB-mediated lysosomal biogenesis; blocks RAB7/LAMP1-dependent phagolysosomal fusion (Paik et al., 2022).	miR-155-5p antagonists; Akt-mTOR inhibitors.
SL-1 (Sulfolipid)	Rv3821 and Rv3822 (Seeliger et al., 2012)	Inhibits mTORC1 to activate TFEB, enhancing lysosomal function; promotes phagosomal acidification (Sachdeva et al., 2020).	SL-1 biosynthesis enzyme inhibitors
1-TbAd	Rv3377c and Rv3378c (Mayfield et al., 2024)	Elevates lysosomal pH to inactivate hydrolases; blocks autophagosome maturation (Bedard et al., 2023).	1-TbAd synthase inhibitors.
Rv1096	Rv1096	Inhibits phagosomal maturation (Deng et al., 2020).	Targeted degradation of Rv1096.
PPK-1	Rv2984 and Rv293232c (Singh et al., 2016)	Disrupts PDIM biosynthesis to enhance phagolysosomal fusion; synergizes with isoniazid/bedaquiline (Chugh et al., 2024).	PPK-1 inhibitors [e.g., raloxifene (Chugh et al., 2024)].
Coronin 1	None (it is a protein in macrophage)	Activates cAMP–cofilin1 axis to block phagosomal trafficking; inhibits lysosomal fusion (Saha et al., 2021).	Coronin 1-cAMP interaction inhibitors.
PknG	Rv0410c (Wang et al., 2021)	Inhibits V-ATPase to impair lysosomal acidification; destabilizes lysosomal membranes (Ge et al., 2022).	PknG kinase inhibitors; lysosomal acidification enhancers.

## 3 The defense mechanisms of lysosomes against Mtb

### 3.1 Regulating the homeostasis of lysosomes

**Figure 2:**
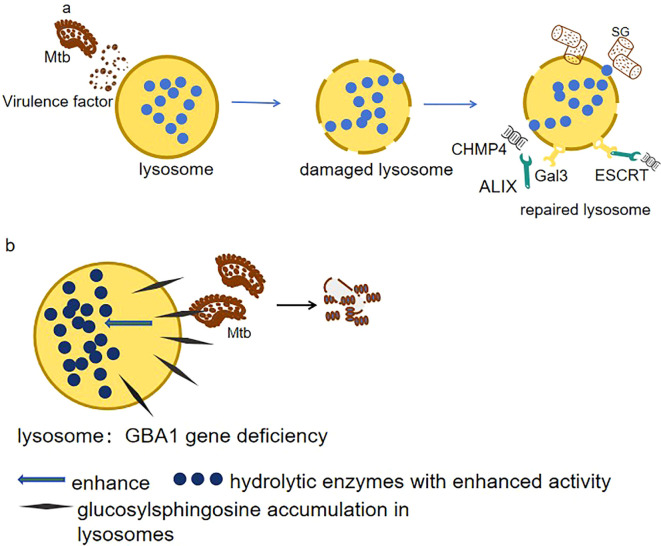
**(a)** Membrane damage resolution. Mtb disrupts lysosomal integrity via ESX-1 secretion system. Host cells counteract this by mobilizing two repair pathways: 1. ESCRT machinery (ALIX/CHMP4): Mediates membrane scission and vesicle shedding to restore lysosomal continuity. 2. Galectin-3 (Gal3)–ubiquitin axis: Recognizes exposed glycans on damaged membranes, recruiting TRIM16 ubiquitin ligase to tag compromised lysosomes for selective autophagy (lysophagy). These coordinated responses inhibit Mtb proliferation by maintaining lysosomal bactericidal capacity. **(b)** Metabolic reprogramming in GBA1 deficiency. Loss of GBA1 elevates glucosylsphingosine (GlcSph) levels in lysosomes, which paradoxically 1. Enhances hydrolase activity: GlcSph stabilizes lysosomal enzymes (e.g., cathepsins), boosting proteolytic degradation of Mtb. 2. Disrupts lipid hijacking: Counters Mtb’s strategy to exploit MMGT1-GPR156 lipid droplet axis for survival.

Lysosomal damage is a feature in many diseases and hinders cellular health. Inner membrane damage causes organelle instability. How cells stabilize the damaged inner membrane for repair is unclear. Current research shows that under various stresses, cells form stress granules. Stress granules modulate mRNA stability and translation to help cells cope with adverse environments (Protter and Parker, 2016). When host cells are infected with Mtb, lysosomal inner membrane is damaged. Stress granules form at the damage site, prevent further membrane rupture, and assist lysosome repair. They can inhibit Mtb proliferation and spread (Bussi et al., 2023). Simultaneously, Galectin-3 (Gal3), which is involved in processes such as cell adhesion, apoptosis, immune response, and autophagy, recognizes the glycans exposed after lysosomal inner membrane damage and recruits ESCRT components (e.g., ALIX and CHMP4) to promote the repair process (Jia et al., 2020b). The dual repair mechanisms of Mtb-induced lysosomal membrane damage—stress granule stabilization and Galectin-3/ESCRT-mediated remodeling and their role in suppressing bacterial proliferation are shown in (**Figure 2a**). Therefore, modulating stress granule formation or associated pathways can develop treatment strategies, targeting pathogen and host cell mechanisms to enhance treatment effectiveness and address drug resistance.

Except for lysosomal damage, lysosomes have abnormal functions related to various diseases like Gaucher disease (GD), which is an autosomal recessive genetic disorder classified under lysosomal storage diseases, and is caused by mutations in the GBA1 gene, disrupting glucocerebrosidase in lysosomes, leading to accumulation and dysfunctions (Andrzejczak and Karabon, 2024). However, it is surprising to see that zebrafish with GBA1 deletion showed resistance to infections by Mtb. GBA1 mutations confer resistance to mycobacterial infections in zebrafish by enhancing lysosomal bactericidal activity through glucosylsphingosine accumulation in macrophage lysosomes (as shown in **Figure 2b**). This lysosomal metabolite exerts bactericidal effects either by modifying membrane properties or by activating enzymatic pathways that potentiate macrophage microbicidal capacity (Fan et al., 2023).

### 3.2 Affect autophagy

Autophagy is an evolutionarily conserved process where cells employ lysosomes to selectively degrade damaged, senescent, or superfluous biomacromolecules and organelles, with the breakdown products recycled to fuel metabolism and facilitate organelle renewal. This lysosome-mediated degradation pathway serves as a fundamental cellular quality control system (Mizushima and Komatsu, 2011). The autophagic cascade consists of crucial steps: initiation, isolation membrane formation, autophagosome generation, and its fusion with lysosomes, ending in degradation and recycling (Mariño et al., 2014). The regulation of autophagy can be achieved through the following key factors: (1) mTOR (mechanistic target of rapamycin): functions as a central nutrient sensor and master autophagy regulator. In nutrient-abundant conditions, it phosphorylates proteins such as the ULK1 complex, inhibiting the initiation of autophagy. However, in situations of starvation, hypoxia, stress, or under the influence of drugs, the activity of mTOR decreases, triggering autophagy (Kim et al., 2011). (2) The AMPK (AMP-activated protein kinase) signaling pathway dominates cellular energy homeostasis. Sensing ATP/AMP ratio, it kicks in when energy wanes (Hardie et al., 2012). Once activated, it curbs mTOR and jump-starts autophagy via the ULK1 (Unc-51 like autophagy activating kinase 1) complex and downstream effectors (Kim et al., 2011). (3) Beclin-1 and the PI3K (phosphatidylinositol 3-kinase) complex: Beclin-1 and the PI3K complex are pivotal in autophagy onset. Their interaction yields PI3P, vital for isolation membrane formation (Tran et al., 2021). (4) TFEB (transcription factor EB): a transcriptional maestro for autophagy and lysosomal biogenesis that relocates to the nucleus during nutrient scarcity or stress to activate both, augmenting intracellular autophagic clearance (Settembre et al., 2011). (5) Oxidative stress: Oxidative stress tweaks autophagy as ROS levels vary. Mild ROS prompts autophagy, and excessive amounts impede and harm cells. Cells deploy antioxidants, e.g., Nrf2, to balance stress and modulate autophagy, safeguarding cell viability in complex settings (Finkel, 2011).

TB infection commonly induces lysosomal dysfunction (Berg et al., 2016; Rombouts and Neyrolles, 2023). Autophagy-related genes (ATGs), in conjunction with their associated proteins and regulatory elements, play pivotal roles in orchestrating cellular responses to lysosomal damage (Cross et al., 2023). Notably, during TB-induced lysosomal damage, membrane Atg8ylation emerges as a central coordinator of stress adaptation. This ubiquitin-like modification directly recruits stress granule (SG) proteins NUFIP2 and G3BP1 to damaged lysosomal membranes, bypassing SG condensate formation. Crucially, NUFIP2 engages the Ragulator-RagA/B complex to suppress mTOR activity, thereby activating autophagy and lysosomal repair programs. These Atg8ylation-driven responses enable cells to survive Mtb infection by restoring lysosomal homeostasis (Jia et al., 2022). When lysosomal membrane repair fails, Gal3 recruits ubiquitin ligases (e.g., TRIM16) to tag damaged lysosomes with ubiquitin signals, initiating lysophagy for their selective autophagic clearance. Concurrently, TFEB activation drives lysosomal biogenesis, ensuring replenishment of functional lysosomes (Jia et al., 2020b). Furthermore, during lysosomal damage, Galectin-9 (Gal9) coordinates AMPK activation by stabilizing ubiquitin signals through enhanced lysosomal glycoprotein binding and dissociation from the deubiquitinase USP9X, thereby driving metabolic and autophagic adaptation (Jia et al., 2020a). This process directly induces autophagy, leading to autophagy-mediated clearance of the membrane-damaged Mtb.

**Figure 3:**
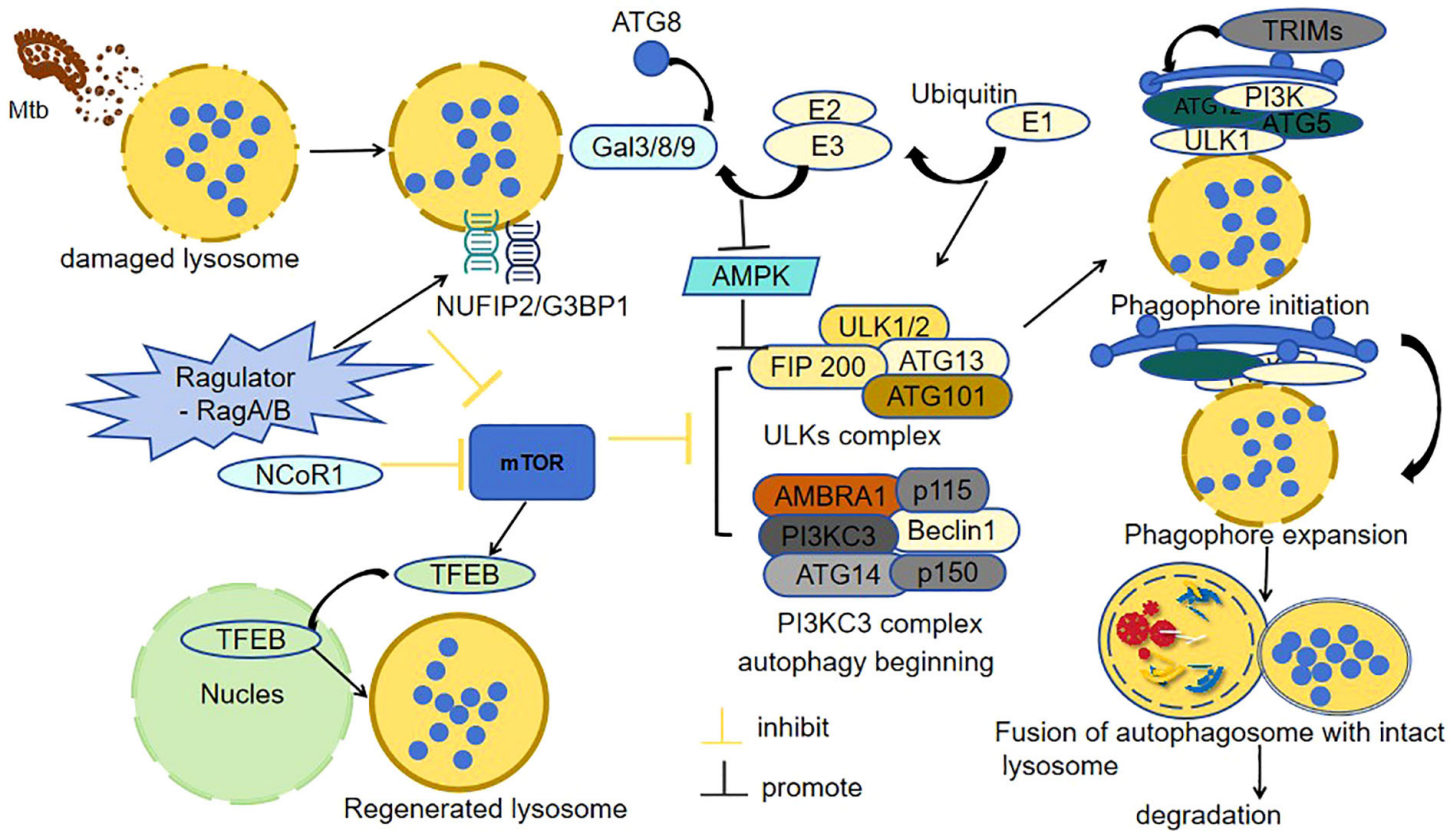
Mtb-induced lysosomal damage and autophagic defense. (Mtb) disrupts lysosomal integrity to evade immune clearance. In response, host cells deploy a coordinated defense: 1. Damage sensing: Galectins (Gal3/8/9) detect lysosomal membrane damage and initiate repair. 2. Autophagy activation: The NUFIP2/G3BP1-Ragulator-RagA/B complex suppresses mTOR, enabling ULK1-mediated autophagosome formation, while AMPK amplifies this signal via energy stress sensing. 3. Lysosomal regeneration: Nuclear translocation of TFEB drives lysosomal biogenesis to replenish functional lysosomes. 4. Quality control: Irreparable lysosomes undergo TRIM-mediated ubiquitination for selective lysophagic degradation. 5. Pathogen clearance: Autophagosomes fuse with intact lysosomes, enabling enzymatic degradation of intracellular Mtb. This integrated response highlights the synergy between lysosomal repair and autophagy in combating TB infection.

During TB infection, the host cell initiates a series of defense mechanisms to combat the invading pathogen. For example, during Mtb infection, NCoR1 (nuclear receptor co-repressor 1) acts as a metabolic-immune checkpoint in myeloid cells, dynamically balancing antibacterial responses through AMPK–mTOR–TFEB axis regulation. Early infection upregulates NCoR1 to activate AMPK, which inhibits mTOR and releases TFEB, driving lysosomal autophagy to restrict bacterial growth. NCoR1 depletion disrupts this pathway, accelerating Mtb proliferation, while AMPK agonists (e.g., AICAR) or mTOR inhibitors (e.g., rapamycin) restore lysosomal function and bacterial clearance (Biswas et al., 2023). In another experiment, an interesting phenomenon has been observed: Mesenchymal stem cells (MSCs) expel anti-TB drugs via host ABC transporters, reducing drug efficacy and promoting Mtb survival. In contrast, Mtb’s own ABC systems primarily import nutrients (Soni et al., 2020). Targeting both host drug-efflux pumps and pathogen nutrient-import pathways may overcome this dual resistance mechanism. However, recent studies by Aqdas et al. have shed light on a potential countermeasure. They discovered that co-stimulating NOD-2/TLR-4 (N2.T4) in mesenchymal stem cells eliminates intracellular Mtb by enhancing lysosomal degradation (3.8-fold Mtb-lysosome fusion↑) and amplifying autophagy through NF-κB/p38 MAPK signaling, achieving 89% bacterial clearance (*p* < 0.001) and highlighting therapeutic potential (Aqdas et al., 2021). This finding strongly suggests that N2.T4 co-stimulation holds great promise as a potential strategy for eradicating Mtb harbored within MSCs, opening up new avenues for TB treatment research. (**Figure 3**) shows the integrated autophagy-lysosomal repair network during Mtb infection, including Atg8ylation-driven stress adaptation, Galectin-mediated lysophagy, and NOD-2/TLR-4 co-stimulation as a therapeutic strategy to enhance bacterial clearance.

### 3.3 Affect phagosomes, lysosomes, and their fusion

Lysosomes are often dubbed the “scavengers” in the body. In the process of lysosomes eliminating foreign substances, the fusion of phagosomes and lysosomes plays a crucial step (Huynh et al., 2007). During phagosome–lysosome fusion, the phagosome’s internal pH is gradually lowered to 4.5 by proton pumps, creating an optimal environment for enzyme-mediated degradation upon fusion with lysosomes, which is regulated by GTPase Rab7 and SNARE proteins such as VAMP8 (Hyttinen et al., 2013). Aylan et al. identified ATG7 and ATG14 as essential regulators of Mtb clearance in human macrophages through compartment-specific autophagy mechanisms. ATG14 drives phagosome–lysosome fusion, limiting phagosomal Mtb survival, while ATG7 enables autophagosome formation to restrict cytosolic replication (Aylan et al., 2023). Mtb subverts macrophage defenses by hijacking the p38 MAPK-CREB signaling axis. Upon infection, Mtb activates transcription factor CREB via the p38 MAPK pathway. This CREB-mediated response suppresses phosphorylation of the necroptotic effectors RIPK3 (receptor-interacting serine) and MLKL (mixed lineage kinase domain-like protein), thereby dampening necroptotic signaling. Consequently, the inhibition of necroptosis disrupts phagolysosome fusion, creating a permissive niche for Mtb to evade lysosomal degradation and sustain intracellular proliferation (Leopold Wager et al., 2023). Considering this, targeting the CREB-mediated pathway with small-molecule inhibitors or modulators could avert the drug resistance risk of direct antibacterial therapies. Additionally, uncovering the specific early genes upregulated by M.tb-induced CREB activation and their functions may reveal novel therapeutic targets. Moreover, SLAMF1 (signaling lymphocytic activation molecule family member 1) serves as a critical regulator of Mtb containment in macrophages. Mtb infection upregulates SLAMF1 expression, which directly enhances bacterial uptake through receptor-mediated phagocytosis. Strikingly, stimulation of SLAMF1 with agonistic antibodies further amplifies this phagocytic capacity. Mechanistically, SLAMF1 colocalizes with Mtb-containing phagosomes and dynamically associates with both early (Rab5) and late (Rab7/LAMP1) endolysosomal markers, driving phagosome maturation toward bactericidal lysosomal compartments. This SLAMF1-dependent endolysosomal reprogramming potently restricts intracellular Mtb survival (Barbero et al., 2021). Similarly, BTLA, an immunoregulatory receptor highly expressed on lymphocytes and macrophages, modulates immune responses through interaction with its ligand HVEM. This BTLA-HVEM signaling axis critically balances immune activation and tolerance to maintain homeostasis (Andrzejczak and Karabon, 2024). Subsequent experiments have indicated that BTLA orchestrates autophagic clearance of Mtb through AKT/mTOR signaling in macrophages. Mtb infection upregulates BTLA expression, which suppresses AKT/mTOR pathway activity to activate autophagic flux. This is evidenced by increased LC3-II conversion and enhanced autophagosome–lysosome fusion, culminating in lysosomal degradation of intracellular bacteria (Liu et al., 2021).

Recent studies reveal that Mtb differentially manipulates lysosomal pathways across host cells to determine infection outcomes. In endothelial cells, Mtb is targeted to acidified phagolysosomes for degradation through itgb3-mediated activation of Rab GTPases, which drive endosomal maturation and autophagic flux (Bussi and Gutierrez, 2019). Conversely, in macrophages and epithelial cells, Mtb suppresses lysosomal acidification by disrupting V-ATPase assembly via virulence factors like LpqH, while coopting the MMGT1-GPR156 lipid droplet axis to sequester antimicrobial lipids and stabilize non-acidified niches (Kalam et al., 2023). These dual strategies—exploiting host ITGB3 (Integrin Subunit Beta 3) for lysosomal clearance in endothelia versus hijacking MMGT1-GPR156 for lipid-dependent persistence in phagocytes—highlight cell type-specific vulnerabilities that could be therapeutically targeted to restore lysosomal bactericidal functions.

While cellular-level research has its own charm, the clinical realm has unveiled a treasure trove of fascinating phenomena that captivate our attention. Deng et al. found that miRNA-215-5p expression was upregulated in patients with TB compared to healthy controls. Upregulation of miRNA-215-5p inhibited autophagy maturation by preventing autophagosome–lysosome fusion (Deng et al., 2023). Thus, regulating miRNA-215-5p may offer a new anti-TB treatment strategy. Further research on its specific regulation of the autophagy pathway may elucidate its role in TB immunopathology. Another study showed that phospholipids and ceramides in the plasma of patients with active TB exhibited significant abnormalities. Notably, lipid metabolic reprogramming emerges as a promising theranostic target in TB. Elevated lysophosphatidic acid species [LPA(16:0/18:0)] in active TB patients’ plasma show perfect diagnostic accuracy (AUC = 1.0), while ceramides enhance antibacterial immunity through autophagy–lysosome coordination, proposing a novel “diagnosis-to-therapy” biomarker strategy (Chen et al., 2021). Thus, targeting lipid metabolism emerges as a novel TB treatment strategy, while lipid biomarkers like LPA serve as dual-purpose biomarkers for diagnosis and treatment monitoring. Furthermore, Mtb accumulates more extracellular polyphosphate than non-pathogenic bacteria, hindering phagocyte killing by inhibiting phagosome acidification and lysosomal activity (Rijal et al., 2020). Genetic deletion of BCG_2432c in the BCG vaccine strain augments autophagy-mediated antimycobacterial immunity through enhanced autophagosome–lysosome fusion and lysosomal proteolytic activity, providing superior protection against Mtb infection compared to parental BCG in preclinical models (Wu et al., 2022).

### 3.4 The communication between lysosomes and other organelles

Lysosomes are crucial intracellular digestive and defensive organelles, and their normal functions are vital for resisting Mtb infection. Lysosomes directly degrade invading pathogens via their hydrolases for preliminary defense and interact with other organelles to combat pathogen invasion and replication (Repnik et al., 2013). For instance, lysosomes and mitochondria maintain cellular homeostasis through reciprocal metabolic exchange: lysosomal metabolites fuel oxidative phosphorylation, while mitochondrial ATP powers lysosomal acidification and function (Deus et al., 2020). Research by Bussi et al. shows that under physiological conditions, lysosomes degrade Mtb through enzymatic activity while exporting metabolites to sustain mitochondrial oxidative phosphorylation; reciprocally, mitochondria generate ATP to power lysosomal acidification and proteolytic functions. However, Mtb disrupts this partnership by compromising lysosomal integrity, triggering cathepsin-mediated degradation of mitochondrial electron transport chain components. The resultant energy crisis forces metabolic reprogramming toward glycolysis, paradoxically enhancing Mtb survival through nutrient enrichment. Therapeutic intervention with α-ketoglutarate restores lysosomal–mitochondrial crosstalk, simultaneously rescuing mitochondrial metabolism and lysosomal bactericidal capacity—demonstrating how leveraging this organelle partnership can overcome pathogen-evolved immunosuppression strategies (Bussi et al., 2022). Moreover, lysosomes and the endoplasmic reticulum (ER) coordinate a multilayered defense against Mtb through dynamic membrane interactions. When Mtb damages lysosomal membranes via its ESX-1, ER-resident oxysterol-binding protein 8 (OSBP8) is recruited to membrane contact sites, where it mediates PI4P-cholesterol exchange to restore lysosomal integrity. This lipid transfer mechanism accomplishes dual protective roles: clearing pathogenic PI4P microdomains to reactivate V-ATPase-driven acidification (pH ≤4.5), and providing cholesterol to stabilize lysosomal membranes against bacterial phospholipase assaults. Concurrently, ER-derived calcium signaling enhances lysosomal protease activation, enabling efficient bacterial degradation. Disruption of this partnership (e.g., OSBP8 deficiency) triggers PI4P overload (>3-fold increase), elevates lysosomal pH (≥6.2), and reduces bacterial killing efficacy by 4.7-fold (Anand et al., 2023). Therapeutic strategies targeting this collaborative axis—such as small molecules enhancing ER-lysosome lipid flux—could counteract Mtb’s membrane sabotage tactics, positioning organelle cooperation as a frontier in host-directed TB therapies.

## 4 Drug research on tuberculosis

### 4.1 Drug repurposing

Although drugs like bedaquiline and delamanid are clinically used for DR-TB, no lysosome-targeted anti-TB drugs have been developed or widely applied thus far. Notably, tamoxifen, widely recognized as a breast cancer drug, has recently been reported to exhibit a direct antibacterial effect on Mtb and to synergize with first-line TB antibiotics (Chen et al., 2014; Jang et al., 2015). In Boland et al.’s study of the zebrafish *Mycobacterium marinum* infection model, tamoxifen’s anti-mycobacterial activity was shown to be independent of its established estrogen receptor pathway function. Instead, it appears to enhance lysosomal function, facilitating the transport of mycobacteria to lysosomes for degradation, thereby suggesting a host-directed mechanism centered on lysosomal modulation (Boland et al., 2023). However, whether tamoxifen directly targets TFEB or LC3 to enhance lysosomal function and promote mycobacterial trafficking to lysosomes for degradation requires further investigation into the molecular mechanisms. These findings especially support the repurposing of tamoxifen for HDT in the treatment of drug-resistant infections, but studies have shown that long-term tamoxifen use significantly increases risks of endometrial cancer, venous thromboembolism (VTE), and non-alcoholic fatty liver disease (NAFLD) (Davies et al., 2013). Moreover, ibrutinib was proved to increase the colocalization of the autophagy marker LC3b with Mtb, promotes the fusion of autophagy–lysosomes, and significantly inhibits the intracellular growth of Mtb. It revealed that ibrutinib increases autophagy in Mtb-infected macrophages by inhibiting the BTK/Akt/mTOR pathway (Hu et al., 2020). However, ibrutinib use may cause cardiotoxicity (e.g., atrial fibrillation) and increase the risk of bleeding and opportunistic infections (Byrd et al., 2013; Yin et al., 2017; Buck et al., 2023). Singh et al. also found that all-trans retinoic acid (ATRA) can enhance the acidification and increase the number of lysosomes by activating the MEK/ERK and p38 MAPK signaling pathways and upregulating the expressions of lysosome-related genes (such as TFEB) (Singh et al., 2024). These changes effectively promote the phagocytosis and elimination of Mtb by cells. Adding ATRA to the standard TB treatment may help clear bacteria more quickly. Therefore, it is necessary to conduct further research on its potential for human use. Additionally, the hepatotoxicity, cutaneous-mucosal toxicity, and increased intracranial pressure associated with ATRA administration must be considered (Tallman et al., 2002). Heemskerk and others found that two antipsychotic drugs, fluspirilene and pimozide, can inhibit the growth of Mtb. On the one hand, these two drugs mainly enhance autophagy by inhibiting calmodulin activity and blocking the Dopamine D2 Receptor (D2R), which promotes more bacteria to be localized in autophagolysosomes. On the other hand, they both can increase the presence of TFEB in the nucleus, thereby enhancing the lysosomal response. In addition, pimozide can also reduce the degradation of lysosomal V-ATPase mediated by cytokine-inducible SH2-containing protein (CISH) induced by Mtb, thus enhancing the acidification of lysosomes and ultimately effectively inhibiting the growth of Mtb (Heemskerk et al., 2021). These two approved antipsychotic drugs are promising candidates for HDT against drug-resistant Mtb infections (Zumla et al., 2016). While specific clinical data are currently lacking, neurotoxicity and cardiotoxicity should be monitored during use (Frangos et al., 1978; Shi et al., 2015). 4-BOP (4-(benzyloxy)phenol) can be found in cosmetics or food packaging materials and acts as an antioxidant to prevent product deterioration due to oxidation. It can activate p53 expression by inhibiting its interaction with KDM1A, resulting in the generation of reactive oxygen species (ROS) and an increase in intracellular calcium levels. These changes trigger phagosome–lysosome fusion and enhance the killing effect on intracellular mycobacteria (Naik et al., 2024). However, 4-(benzyloxy)phenol may have potential carcinogenicity and cytotoxicity at high concentrations.

### 4.2 Phytochemicals

Clinical chemical drugs occupy a pivotal position in contemporary medicine; however, recent research has progressively concentrated on plant-derived compounds, termed phytochemicals, owing to their potential therapeutic advantages, such as epigallocatechin gallate, abbreviated as EGCG, which is a polyphenolic compound that widely exists in nature and has important biological activities. It is especially abundant in green tea. However, clinical studies have shown that daily intake exceeding 600 mg may increase the risk of liver damage, and after oral administration, it is easily and rapidly biotransformed and degraded in the gastrointestinal tract and liver, resulting in low bioavailability and ultimately limiting its clinical application effectiveness (Dekant et al., 2017; Peng et al., 2024). It can downregulate TACO gene transcription, weakening TACO’s inhibition of phagosome–lysosome fusion. The activation of AMPK inhibits mTORC1 activity, thereby relieving its suppression on ULK1 and initiating the formation of autophagosome precursors. Moreover, it promotes TFEB nuclear translocation and induces mitochondrial ROS generation and activates the MAPK/ERK pathway to upregulate the expression of V-ATPase subunits and enhance the acidic environment within lysosomes. Moreover, compared with oral administration, pulmonary delivery of EGCG could enhance drug concentration in the lungs, avoid metabolism, and reduce systemic side effects (Sharma et al., 2020). Another is curcumin, a natural polyphenolic compound extracted from the rhizomes of *Curcuma longa* of the Zingiberaceae family, which has remarkable anti-inflammatory, antioxidant, anti-tumor, antibacterial, and antiviral effects (Chen et al., 2018; Eke-Okoro et al., 2018). Owing to its diverse biological activities, curcumin has garnered extensive attention in the fields of medicine and healthcare. Similarly, curcumin enhances autophagy through the AMPK/mTOR/ULK1 pathway, downregulates TACO expression, activates Rab7, and upregulates LAMP1 to promote phagosome–lysosome fusion, while further promoting TFEB (transcription factor EB) nuclear translocation and upregulating V-ATPase subunit expression driving lysosomal acidification and thereby clearing intracellular Mtb (Gupta et al., 2023). As a natural compound, curcumin has relatively low toxicity and may offer good safety in clinical applications. In the context of the growing problem of DR-TB, it presents a new option for treatment.

### 4.3 Nanomedicines

Nanomaterials, as an emerging class of materials, are being actively explored for their unique properties and promising applications across various fields, including medicine, where they hold potential for novel therapeutic and diagnostic approaches. Gupta et al. demonstrated that ISCurNP significantly reduced the CFU count in Mtb-infected RAW cells, and the synergistic combination of ISCurNP with isoniazid markedly enhanced Mtb killing (Gupta et al., 2023). ISCurNP not only targets macrophages and boosts curcumin’s bioavailability but also chelates free iron within lysosomes to block the iron uptake pathway of Mtb, thereby inhibiting its growth. This dual mechanism effectively combats Mtb, offering new therapeutic possibilities for TB, particularly in drug-resistant cases. Similarly, Se NPs and Ison@Man-Se NPs preferentially accumulate in macrophages where they activate AMPK, inhibit mTORC1 to initiate autophagosome formation, and upregulate LAMP1 to promote phagosome–lysosome fusion, thereby enhancing the clearance of intracellular Mtb (Pi et al., 2020).

Regarding other aspects, YDGP is a porous microparticle derived from the yeast cell wall. It serves as a targeted drug delivery system to macrophages by binding to surface receptors Dectin-1. This binding initiates NADPH oxidase-dependent ROS production and regulates the LC3-associated autophagy pathway, which jointly facilitate phagosomal maturation. Through these synergistic mechanisms, YDGP not only improves the anti-TB effect by clearing intracellular pathogens but also offers a strategic framework for developing immunotherapeutic agents targeting macrophage-mediated immunity (Fatima et al., 2021). Similarly, Ahmad et al. demonstrated that rifabutin-loaded β-glucan microparticles (DYDGP) targeted macrophage Dectin-1 receptors to activate NOX2-dependent ROS burst and LC3-II-mediated macroautophagy. This dual mechanism enhanced phagolysosomal fusion (a 2.7-fold increase in LAMP-1 expression) and autophagosome maturation (an 83% enhancement in AVO formation), achieving a 3.1-log reduction in CFU counts of multidrug-resistant MDR-M.tb strains (Ahmad et al., 2024). Although YDGP exhibits excellent biocompatibility as a natural polysaccharide, prolonged administration at high doses may lead to excessive immune activation, oxidative tissue damage, and cellular toxicity. Consequently, meticulous dose optimization and functional modifications such as ligand conjugation are essential to ensure optimal therapeutic safety and efficacy.

### 4.4 Probiotic preparations

Alongside the emerging field of nanomaterials, microorganisms are indispensable to life, with their diverse functions ranging from nutrient cycling to disease pathogenesis, making them a central focus of scientific inquiry and biotechnological innovation. Rahim et al. demonstrated that PMC203 (*Lactobacillus rhamnosus*) significantly induces autophagy and lysosomal biosynthesis, thereby reducing Mtb load in macrophages (Rahim et al., 2024). Although the exact mechanisms remain to be elucidated, it may involve gene expression regulation or other pathways. These findings underscore the potential of probiotic-mediated autophagy as a novel therapeutic approach for TB. Although *in vitro* studies have demonstrated its favorable safety profile, further *in vivo* animal studies remain needed to confirm its efficacy and safety (Rahim et al., 2024). These findings are summarized in (**Table 2**), which highlights candidate therapeutics – including repurposed drugs, phytochemicals, nanomedicines, and probiotics – that target lysosomal-autophagy pathways to eliminate Mtb, along with their mechanistic insights and clinical translational challenges.

**Table 2:** Potential drugs for treating Mtb.

Mechanism	Drugs	Potential Targets	Mode of Action
Lysosomal Function Enhancement			
TFEB-mediated biogenesis	Tamoxifen	TFEB, LC3	Promotes lysosomal trafficking and bacterial degradation via ER-independent pathways (Boland et al., 2023).
Lysosomal acidification	4-BOP	p53-KDM1A, V-ATPase	Induces ROS/calcium signaling to activate lysosomal acidification (Naik et al., 2024).
Phagosome–Lysosome Fusion			
TACO suppression	EGCG	TACO, AMPK/mTOR/ULK1	Downregulates TACO; activates AMPK/mTOR/ULK1-TFEB axis (Sharma et al., 2020).
Rab7/LAMP1 activation	Curcumin	Rab7, LAMP1, V-ATPase	Enhances vesicular trafficking and acidification via TFEB-driven V-ATPase upregulation (Gupta et al., 2023).
Calmodulin/D2R blockade	Fluspirilene/Pimozide	Calmodulin, D2R, CISH	Stabilizes V-ATPase by inhibiting CISH-mediated degradation (Heemskerk et al., 2021).
Autophagy Regulation			
mTOR pathway inhibition	Ibrutinib	BTK/Akt/mTOR, LC3	Enhances LC3-II-dependent autophagosome–lysosome fusion (Hu et al., 2020).
Iron chelation	ISCurNP	Lysosomal iron transport	Depletes iron availability while boosting autophagy flux (Pi et al., 2020).
Dectin-1/LC3 activation	YDGP/DYDGP	Dectin-1, NOX2, LC3	Triggers NADPH oxidase-dependent ROS and LC3-II-mediated phagosomal maturation (Fatima et al., 2021).
Probiotic-Induced Autophagy			
TFEB pathway activation	PMC203	TFEB	Induces lysosomal biogenesis through undefined autophagy initiation mechanisms (Rahim et al., 2024).

## 5 Conclusions and prospects

Researching on the interaction mechanism between TB and lysosomes, significant progress has been achieved. Numerous mycobacterial factors affecting lysosomal impact have been identified. These findings provide crucial insights into TB pathogenesis. However, current research faces limitations; some results remain inconsistent or contradictory. Future studies require more precise and comprehensive methods, such as advanced imaging and molecular biology techniques, to better monitor the dynamic interaction between lysosomes and Mtb for obtaining more reliable data.

The defense mechanisms of lysosomes against Mtb exhibit multifaceted characteristics. Studies on lysosome–mitochondria communication have uncovered novel pathways for intracellular material transport and metabolic regulation, informing new treatment strategies based on organelle interactions. Future research should explore both the synergistic effects among these mechanisms and their variations during different infection stages and cell types.

In the realm of TB drug research, while certain drugs have demonstrated some anti-TB potential, they still encounter numerous formidable challenges in widespread clinical applications. However, the efficacy, safety, and long-term stability of these drugs and formulations require rigorous validation through large-scale, multi-center clinical trials. Therefore, future drug development efforts should focus on optimizing drug structures and formulations, significantly improving drug targeting and bioavailability, and actively exploring rational and effective combination therapies to effectively address TB drug resistance and enhance treatment efficacy.

The strategy of targeting lysosomes for TB treatment holds great application prospects and is anticipated to overcome many limitations of traditional TB treatment modalities. By implementing a series of effective measures, such as enhancing lysosomal function and adopting advanced lysosome-targeted drug delivery systems, the treatment outcomes of Mtb and drug-resistant Mtb can be significantly improved. Nevertheless, this strategy still faces multiple challenges in clinical application, including the optimization of drug design, in-depth exploration of the mechanism of action, the formulation of personalized treatment regimens, and clinical translation.

With the continuous advancement of science and technology and deeper interdisciplinary collaboration, it is expected that in the foreseeable future, the strategy of targeting lysosomes for TB treatment will achieve significant breakthroughs, bringing new hope for improving the treatment outcomes and quality of life of patients with TB.
